# Prosthetic model, but not stiffness or height, affects maximum running velocity in athletes with unilateral transtibial amputations

**DOI:** 10.1038/s41598-019-56479-8

**Published:** 2020-02-04

**Authors:** Paolo Taboga, Emily K. Drees, Owen N. Beck, Alena M. Grabowski

**Affiliations:** 1 California State University, Sacramento, CA USA; 2 University of Colorado Boulder, Boulder, CO USA; 3 George W. Woodruff School of Mechanical Engineering, Georgia Institute of Technology, Atlanta, GA USA; 4 School of Biological Sciences, Georgia Institute of Technology, Atlanta, GA USA; 5 VA Eastern Colorado Healthcare System, Denver, CO USA

**Keywords:** Bone quality and biomechanics, Musculoskeletal system

## Abstract

The running-specific prosthetic (RSP) configuration used by athletes with transtibial amputations (TTAs) likely affects performance. Athletes with unilateral TTAs are prescribed C- or J-shaped RSPs with a manufacturer-recommended stiffness category based on body mass and activity level, and height based on unaffected leg and residual limb length. We determined how 15 different RSP model, stiffness, and height configurations affect maximum running velocity (v_max_) and the underlying biomechanics. Ten athletes with unilateral TTAs ran at 3 m/s to v_max_ on a force-measuring treadmill. v_max_ was 3.8–10.7% faster when athletes used J-shaped versus C-shaped RSP models (p < 0.05), but was not affected by stiffness category, actual stiffness (kN/m), or height (p = 0.72, p = 0.37, and p = 0.11, respectively). v_max_ differences were explained by vertical ground reaction forces (vGRFs), stride kinematics, leg stiffness, and symmetry. While controlling for velocity, use of J-shaped versus C-shaped RSPs resulted in greater stance average vGRFs, slower step frequencies, and longer step lengths (p < 0.05). Stance average vGRFs were less asymmetric using J-shaped versus C-shaped RSPs (p < 0.05). Contact time and leg stiffness were more asymmetric using the RSP model that elicited the fastest v_max_ (p < 0.05). Thus, RSP geometry (J-shape versus C-shape), but not stiffness or height, affects v_max_ in athletes with unilateral TTAs.

## Introduction

**Figure 1:**
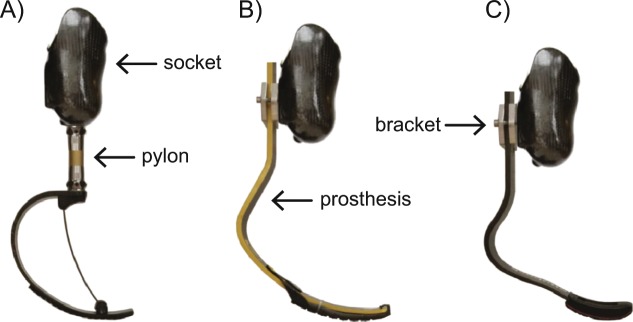
(**A**) Freedom Innovations Catapult (FDM), (**B**) Össur Cheetah Xtend (OSR), and (**C**) Ottobock 1E90 Sprinter (OBK) running-specific prosthetic models attached in-series to a carbon fiber socket that encompasses the residual limb. The height of the prosthesis is adjusted using a pylon or height adjustment bracket.

Running-specific prostheses (RSPs) are passive-elastic devices typically made of carbon fiber that attach to a socket that surrounds the residual limb. The use of RSPs enable athletes with transtibial amputations (TTAs) to compete in running events including the Olympic games. RSP models are generally C-shaped or J-shaped. C-shaped RSPs attach distal to the socket and are recommended for distance running^1^ (e.g. 10 km, half marathon, and marathon) and J-shaped RSPs attach posterior to the socket and are recommended for sprinting^1^ (e.g. 100 m, 200 m, and 400 m). Despite different attachments and shapes, both types of RSPs act in-series with the residual limb. Athletes with TTAs are prescribed an RSP with a manufacturer-recommended stiffness category that is based on his or her body mass and activity level^1–3^. Greater stiffness categories correspond with stiffer RSPs while considering prosthetic model^4^. Further, for an athlete with a unilateral TTA, RSP height is set based on the athlete’s contralateral unaffected leg length, stride kinematics, and their prosthetist’s and personal preference^5^. The height of a C-shaped RSP is adjusted by shortening or lengthening the pylon that connects the RSP to the socket, while the height of a J-shaped RSP is adjusted by changing its mounting position posterior to the socket (Fig. 1). An athlete’s unloaded RSP height is adjusted so that their affected leg (AL) length is 2–8 cm taller than their unaffected leg (UL) length^6,7^. The RSP configuration (i.e. model, stiffness category, height) used by athletes with TTAs likely affects their running performance, which is well-correlated with the maximum running velocity that an athlete can attain^8^.

Running velocity equals the product of stride frequency and stride length, where a stride is two steps and a step is the period of ground contact and subsequent aerial time^7^. Step frequency equals the number of steps in a given period of time, and step length equals the forward distance traveled by the centre of mass (CoM) relative to the ground with each step. At a given velocity, step length can be expressed as the product of the average vertical ground reaction force (GRF) applied during ground contact normalized to body weight and contact length (the product of contact time and forward velocity), which is the forward distance traveled by the CoM during ground contact^9^. Thus, running velocity equals the product of step frequency, stance average vertical GRF, and contact length. Runners increase step frequency by decreasing ground contact time and primarily increase step length by applying greater vertical GRFs on the ground^10^. Greater vertical GRFs increase the vertical CoM velocity at the end of ground contact, resulting in longer aerial time, and thus increasing the forward CoM distance traveled for each step at a given running velocity. However, at a given velocity, an increase in step length or frequency is counteracted by a decrease in the other parameter^10^. For example, an increase in vertical velocity at the end of ground contact would increase step length but also increase step time, which would decrease step frequency, and not change running velocity. Non-amputees and athletes with TTAs exhibit directionally similar changes in stance average vertical GRFs and spatiotemporal variables to increase running velocity, but biomechanics differ between the AL versus UL^6,7^.

The biomechanics of level-ground running are well represented by a spring-mass model^11–14^. In this model, the leg is represented by a massless linear spring, and body mass is represented by a point mass. Leg stiffness equals the peak vertical GRF divided by CoM displacement along the leg. The relationship between leg stiffness and running velocity is unclear: non-amputees either maintain or increase leg stiffness as running velocity increases^6,15,16^. Athletes with unilateral TTAs decrease AL stiffness, while UL stiffness remains constant from 3.0 m/s up to maximum running velocity (v_max_)^6^, thus leg stiffness values between the AL and UL are more asymmetric at faster velocities.

Previous studies found no relationship between biomechanical asymmetries (i.e. differences in spatiotemporal, GRF, and stiffness variables between legs) and sprint performance in non-amputees^17–19^. However, it is possible that the anatomical differences between the legs of an athlete with a unilateral TTA result in larger biomechanical asymmetries compared to non-amputees: for example, reported values of asymmetries in non-amputee sprinters vary between 1.1% (step frequency)^18^ and 1.3% (step length)^18^, and 4.2% (ground contact time)^17^ and 4.9% (aerial time)^17^. On the other hand, previous studies have shown that athletes with unilateral TTAs apply 9% lower stance average vertical GRFs with their AL compared to UL using a typical RSP configuration across a range of speeds up to v_max_^7^. Because step length equals the product of contact length and stance average vertical GRF at a given velocity^9^, if one leg has a limited ability to apply GRFs compared to the other leg, step length will be reduced. If the reduced step length is not compensated by an increase in step frequency, this would limit v_max_. Additionally, athletes with unilateral TTAs have an 8% slower step frequency at v_max_ for their AL compared to their UL, while ground contact time/length does not differ between legs^7^. Further, Beck *et al*.^20^ found that more symmetric peak vertical GRFs between the AL and UL of athletes with unilateral TTAs reduced the metabolic cost of running at 2.5–3.0 m/s. However, the effects of different RSP configurations on biomechanical asymmetry and v_max_ in athletes with unilateral TTAs are not yet fully understood.

Given the subjective nature of RSP prescription, the purpose of our study was to quantify how use of different RSP model, stiffness, and height configurations affect maximum running velocity (v_max_) and the underlying biomechanics in athletes with a unilateral TTA. Due to the lack of quantifiable information about how use of different RSP models affect v_max_, we tested the null hypothesis that prosthetic model would not affect v_max_. Because non-amputees maintain or increase leg stiffness with faster running velocity^6,15,16^, we hypothesized that use of stiffer RSPs would increase v_max_. Due to the tradeoff between step frequency and step length^10^, we tested the null hypothesis that v_max_ would be independent of RSP height. Moreover, we hypothesized that use of the optimal RSP model, stiffness, and height combination would result in faster step frequencies, greater stance average vertical GRFs, longer contact lengths, and the fastest v_max_. Based on the idea that a limitation in one leg can reduce overall performance, we also hypothesized that athletes would exhibit less asymmetric biomechanics (step frequency, stance average vertical GRF, and contact length) using the optimal RSP configuration.

## Methods

### Participants

Ten athletes with a unilateral TTA (Table 1) gave written informed consent prior to participation. The protocol was approved by the Colorado Multiple Institutional Review Board and the United States Army Medical Research and Materiel Command Human Research Protection Office. All research was performed in accordance with relevant guidelines and regulations. Participants reported no cardiovascular, pulmonary, musculoskeletal, or neurological disease or disorder beyond a TTA.

**Table 1:** Subject demographics, 100 m personal record (PR), usual running-specific prosthesis (RSP) and shape, and RSP configuration that resulted in the fastest maximum velocity (v_max_).

Subject	Sex	Age (years)	Height (m)	Mass (kg)	100 m PR (s)	Affected side	Cause of amputation	Usual RSP		Fastest RSP		
								Model	Shape	Model	Cat	Ht (cm)
1	M	23	1.90	82.6	10.75	Right	Traumatic	Össur Xtreme	J	OBK	0	0
2	M	22	1.70	72.4	11.28	Right	Traumatic	Össur Xtreme	J	FDM	0	2
3	M	31	1.84	75.5	11.55	Right	Traumatic	Össur Xtreme	J	OBK	1	0
4	M	33	1.75	67.5	12.41	Left	Traumatic	Össur Xtreme	J	OBK	−1	+1
5	M	33	1.83	92.6	13.8	Right	Traumatic	Freedom Catapult	C	OBK	0	−2
											0	+0.8
6	M	25	1.78	89.2	14.26	Left	Traumatic	Össur Xtreme	J	OBK	1	+2
7	F	21	1.65	60.0	14.58	Left	Traumatic	Össur Cheetah**	J	OBK	−1	0
8	M	37	1.85	91.0	14.58	Right	Traumatic	Freedom Catapult	C	OSR	+1	+2
9	F	29	1.57	58.9	14.69	Left	PVD	Ottobock 1E90	J	OBK	0	0
10	F	27	1.79	76.4	16.3	Left	Cancer	Össur Flex Run	C	OSR	0	0

Cat is the stiffness category: 0 is recommended, +1 and −1 are one category more and less stiff, respectively. Ht is the RSP height in cm, where 0 is the recommended height. Subject 5 reached the same v_max_ using 2 different heights with the same RSP model. Subject 4 and 5 could only achieve +1 cm and +0.8 cm, respectively, because their residual limb length and relative positioning of the RSP attachment were too proximal and the build height of the OBK model was shorter than OSR (Fig. 1).

*PVD: peripheral vascular disease. **modified with an extra carbon fiber strut

Each participant first completed an alignment and accommodation session lasting about 4 hours. A certified prosthetist aligned each participant with three different prosthetic models: Freedom Innovations Catapult FX6 (FDM; Irvine, CA), Ottobock 1E90 Sprinter (OBK; Duderstadt, Germany), and Össur Cheetah Xtend (OSR; Reykjavik, Iceland). We chose these models because we have previously established the mechanical properties for each model^4^. FDM is C-shaped, while OBK and OSR are J-shaped (Fig. 1). Each participant was aligned with the manufacturer’s recommended stiffness category (based on body mass) and ±1 stiffness category, and at the manufacturer’s recommended height and ±2 cm. We adjusted the height of FDM (C-shaped RSP) by changing the height of the pylon connecting the socket to the RSP, and we adjusted the height of OBK and OSR (J-shaped RSPs) using custom aluminum brackets (Fig. 1). Some athletes used two different sockets: one socket for the C-shaped RSP (FDM) and a different socket for the J-shaped RSPs (OBK, OSR). During the accommodation session, participants ran using each RSP configuration on a treadmill at self-selected speeds and the prosthetist made adjustments until both the participant and prosthetist were satisfied.

### Biomechanical measurements

Each athlete performed sets of running trials consisting of at least 8 strides per trial at constant velocities on a 3D force measuring treadmill (Treadmetrix, Park City, UT). Each series of trials began at 3 m/s, rest was provided between trials, and after each successive trial we incremented treadmill velocity by 1 m/s until the athlete approached their v_max_, where we used smaller velocity increments until athletes reached their v_max_. Athletes usually lowered themselves from the handrails onto the moving treadmill belt to initiate each trial; however, some athletes stood on the treadmill belt and accelerated at 1.0 m/s^2^ (treadmill default acceleration that was constant for all athletes) with the treadmill belt until it reached the desired velocity and the trial began. All of the athletes were experienced with treadmill running. v_max_ was defined as the velocity where athletes took at least 8 strides on the treadmill while maintaining their fore-aft position^9^. If an athlete could not maintain the velocity for 8 strides, we allowed them to repeat the trial, after *ad-libitum* rest. For each series of trials, athletes used one of 15 different combinations of RSP model, stiffness, and height. Prosthetic models were FDM, OBK, and OSR. Prosthetic stiffness conditions were the manufacturer recommended stiffness category (based on body mass and high activity level) and ±1 stiffness categories. Prosthetic height conditions were manufacturer recommended height and ±2 cm. We randomized the trial order of RSP model and stiffness category (3 RSP models × 3 stiffness categories = 9 trials). Then for each RSP model, height was only adjusted for the stiffness category that enabled the fastest v_max_. We randomly inserted the different height trials into the trial order (3 RSP models × 2 RSP heights = 6 trials). Participants completed a maximum of 3 series of trials per day to minimize any potential effects of fatigue; thus, the entire protocol required at least 5 days. Depending on the feedback of each participant, additional rest days were taken to further minimize fatigue, and the average duration to complete the protocol was 10 days.

Throughout each trial, we measured 3D GRFs at 1000 Hz and filtered them using a 4^th^ order low-pass Butterworth filter with a 30 Hz cutoff. We used these filtered data and a 30 N vertical GRF threshold to detect ground contact and calculate GRF parameters, step kinematics, and leg stiffness for each leg (AL and UL) during each step using a custom MATLAB script (MathWorks, Natick, MA). We determined step time for each leg as the sum of the ground contact time and subsequent aerial time. We calculated contact length as the product of ground contact time and the treadmill velocity. For each variable (step frequency, stance average vertical GRF, contact length, contact time, aerial time, and leg stiffness), we calculated the average of both legs. Participants ran with reflective markers attached to the distal end of their RSP and the fifth metatarsal head of their UL. We tracked the position of these markers at 200 Hz (Vicon Nexus, Oxford, UK), filtered marker position data using a 4^th^ order low-pass Butterworth filter with a 7 Hz cutoff, and used a custom MATLAB script to confirm treadmill belt velocity during each foot- or RSP-ground contact.

We used the mean AL peak resultant GRFs measured in the current study and the reported RSP stiffness values from each device reported by Beck *et al*.^4^ (Supplementary Tables 1, 3 and 4) (k_RSP_) to calculate RSP displacement (ΔRSP):

1 $$\Delta {\rm{RSP}}=\frac{Peak\,GRF}{{k}_{RSP}}$$

We calculated leg stiffness (k_leg_) in kN/m as the ratio between peak vertical GRF and maximum leg displacement (ΔL) during each stance phase for the respective limb:

2 $${k}_{leg}=\frac{Peak\,vGRF}{\Delta L}$$

To determine ΔL, we calculated the angle of the leg (relative to vertical) at initial ground contact (contact angle, θ) using running velocity (v), ground contact time (t_c_), and initial leg length (L_0_) for each leg:

3 $$\theta ={\sin }^{-1}(\frac{v{t}_{c}}{2{L}_{0}})$$

We measured initial UL length (L_0_) as the distance from the greater trochanter to the floor during standing, and AL length (L_0_) as the distance from the greater trochanter to the distal end of the unloaded RSP^6,7,21^. We calculated maximum vertical displacement of the CoM (Δy) by twice integrating the vertical acceleration of the CoM with respect to time^22^. Then, we used Δy, L_0_, and θ to calculate ΔL according to McMahon and Cheng^12^:

4 $$\Delta L=\Delta y+{L}_{0}(1-\,\cos \,\theta )$$

We also calculated the symmetry index (SI) for all biomechanical variables of the AL and UL as a ratio between legs, according to Robinson *et al*.^23^:

5 $$SI=\frac{va{r}_{AL}-va{r}_{UL}}{0.5\,(va{r}_{AL}+va{r}_{UL})}$$

A positive SI means that the value for the AL is greater than the value for the UL, while a negative SI means that the value for the AL is lower than the value for the UL. An SI of zero indicates perfect symmetry between the AL and UL.

### Statistical analysis

We used a linear mixed model to analyze the influence of RSP model, stiffness category, and height on v_max_. We used a second linear mixed model to analyze the influence of RSP model, actual stiffness (in kN/m), and height on v_max_. We used additional linear mixed models to analyze the influence of significant RSP configurations (model, stiffness, and/or height) on both legs’ average and SI of biomechanical variables (step frequency, stance average vertical GRF, contact length, contact time, aerial time, and leg stiffness) while accounting for differences in v_max_. We report the fixed effect (β) from each statistically significant association (dependent variable = β independent variable + intercept). We selected linear mixed models, as opposed to simple linear regression analyses, to control for subject variability. Each subject was classified as a random effect, while the independent variables were classified as fixed-effect variables. Linear mixed models are particularly useful in a repeated measures design because they take into account the lack of independence between observations within the same athlete using different RSP configurations^24^. For all linear mixed models, RSP model was classified as a categorical variable, while the stiffness category, actual stiffness, height and all biomechanical variables were classified as continuous variables. We also used one-sample t-tests to compare SI values to perfect symmetry between legs (SI = 0). We used a significance level of p < 0.05. When applicable, we implemented Bonferroni corrections to account for multiple comparisons. We performed all statistical tests using RStudio (RStudio Inc., Boston, MA).

## Results

**Figure 2:**
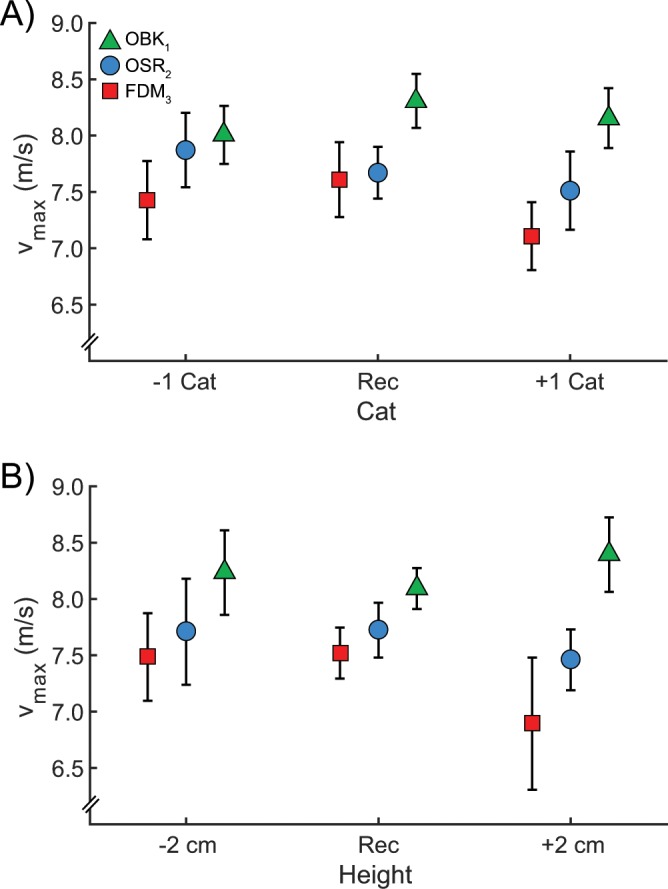
Average ± SEM maximum velocity (v_max_) when athletes used the Ottobock 1E90 Sprinter (OBK_1_), Össur Cheetah Xtend (OSR_2_), and Freedom Innovations Catapult (FDM_3_) running-specific prosthetic (RSP) models with (**A**) stiffness categories (Cat) that are recommended (Rec), one Cat less stiff (−1) and one Cat more stiff (+1) than Rec, and (**B**) heights that are recommended (Rec), two cm shorter (−2) and two cm taller (+2) than Rec height. There was no effect of stiffness category or height on v_max_. Use of the OBK_1_ RSP resulted in the fastest v_max_, followed by use of OSR_2_, and use of FDM_3_ RSPs.

The v_max_ (avg ± SD) for athletes with unilateral TTAs using OBK, OSR, and FDM RSPs were 8.18 ± 1.00 m/s, 7.67 ± 1.25 m/s, and 7.39 ± 1.30 m/s, respectively. Thus, RSP models will be referred to as OBK_1_, OSR_2_, and FDM_3_ to denote order based on v_max_ reached when using each model (1 = fastest, 2 = middle, 3 = slowest). Compared to use of the C-shaped FDM_3_ RSP, v_max_ was 10.7% (β_1_ = 0.82, p < 0.05) and 3.8% (β_2_ = 0.36, p < 0.05) faster when athletes with unilateral TTAs used J-shaped OBK_1_ and OSR_2_ RSPs, respectively. Additionally, use of an OBK_1_ RSP resulted in 6.6% (β_1_ = 0.46, p < 0.05) faster v_max_ compared to use of an OSR_2_ RSP. RSP stiffness category, actual stiffness, and height did not influence v_max_ (p = 0.72, p = 0.37, and p = 0.11, respectively; Fig. 2) and there were no significant interactions among RSP model, stiffness, and height for v_max_ (p > 0.05).

**Figure 3:**
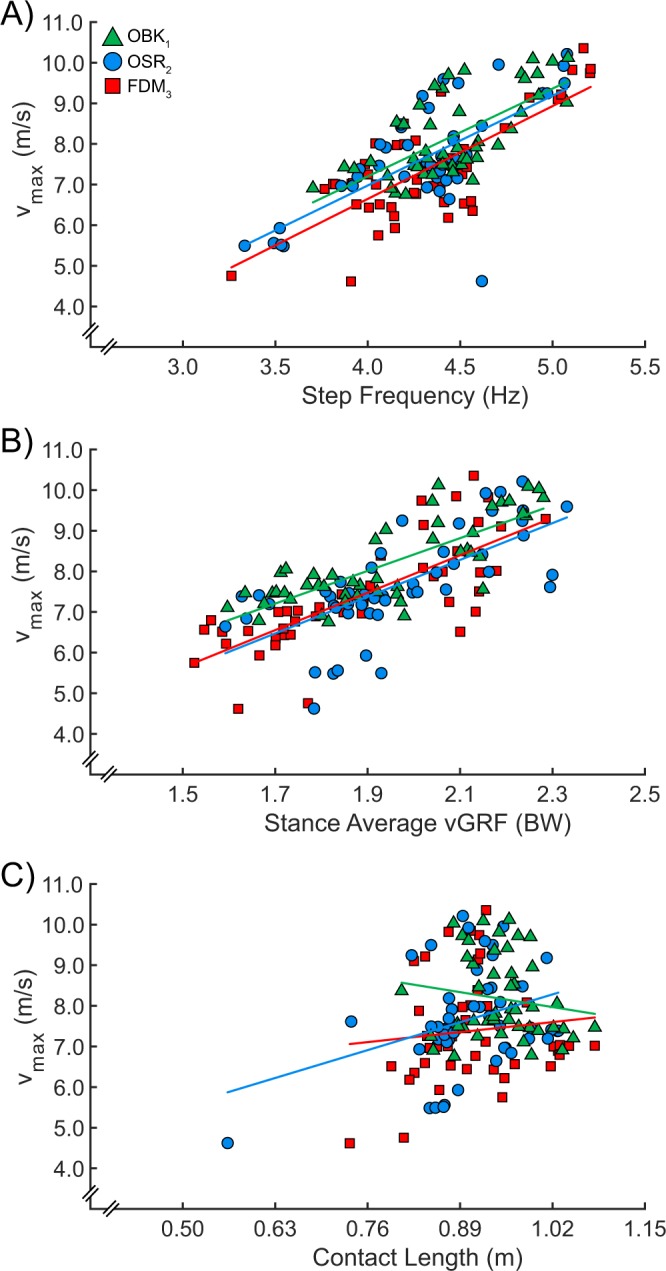
Maximum velocity (v_max_) as a function of (**A**) step frequency, (**B**) stance average vertical ground reaction force (vGRF), and (**C**) contact length when using Ottobock 1E90 Sprinter (OBK_1_), Össur Cheetah Xtend (OSR_2_), and Freedom Innovations Catapult (FDM_3_), running-specific prosthetic models. Each individual data point represents the average value between the affected and unaffected legs for a single trial. Trend lines represent linear regressions for each prosthetic model. Coefficients of determination for linear regressions are shown in Table 2.

When accounting for differences in v_max_, we found that use of FDM_3_ resulted in 3.4% faster step frequencies than use of OBK_1_ and OSR_2_ RSPs (β_1_ = −0.088 and β_2_ = −0.088, both p < 0.05; Fig. 3A, Table 2), and the step frequencies were non-different between use of OBK_1_ and OSR_2_ RSPs (p = 0.99). Use of OSR_2_ resulted in 2.3% and 4.6% greater stance average vertical GRF than use of OBK_1_ and FDM_3_ RSPs, respectively (β_1_ = 0.040 and β_3_ = 0.080, both p < 0.05; Fig. 3B, Table 2), and use of OBK_1_ resulted in 2.3% greater stance average vertical GRF than use of FDM_3_ RSPs (β_3_ = 0.040, p < 0.05). Use of OBK_1_ and FDM_3_ RSPs resulted in 4.4% longer contact lengths than use of OSR_2_ (β_1_ = 0.021 and β_3_ = 0.021, both p < 0.05; Fig. 3C, Table 2), but there was no significant difference in mean contact length between use of OBK_1_ and FDM_3_ RSPs (p = 0.98).

**Table 2:** Coefficients of determination (R-squared) for statistically significant (p < 0.05) linear regressions between biomechanical variables and maximum velocity (Figs. 3 and 4) when using Ottobock 1E90 Sprinter (OBK_1_), Össur Cheetah Xtend (OSR_2_), and Freedom Innovations Catapult (FDM_3_) running-specific prosthetic models. - indicates the biomechanical variable had no effect on maximum running velocity.

	R-squared		
	OBK_1_	OSR_2_	FDM_3_
Step frequency	0.45	0.49	0.57
Stance average vertical ground reaction force	0.61	0.47	0.58
Contact length	—	0.11	—
Contact time	0.81	0.72	0.80
Aerial time	0.10	—	—
Leg stiffness	0.38	0.20	0.10

**Figure 4:**
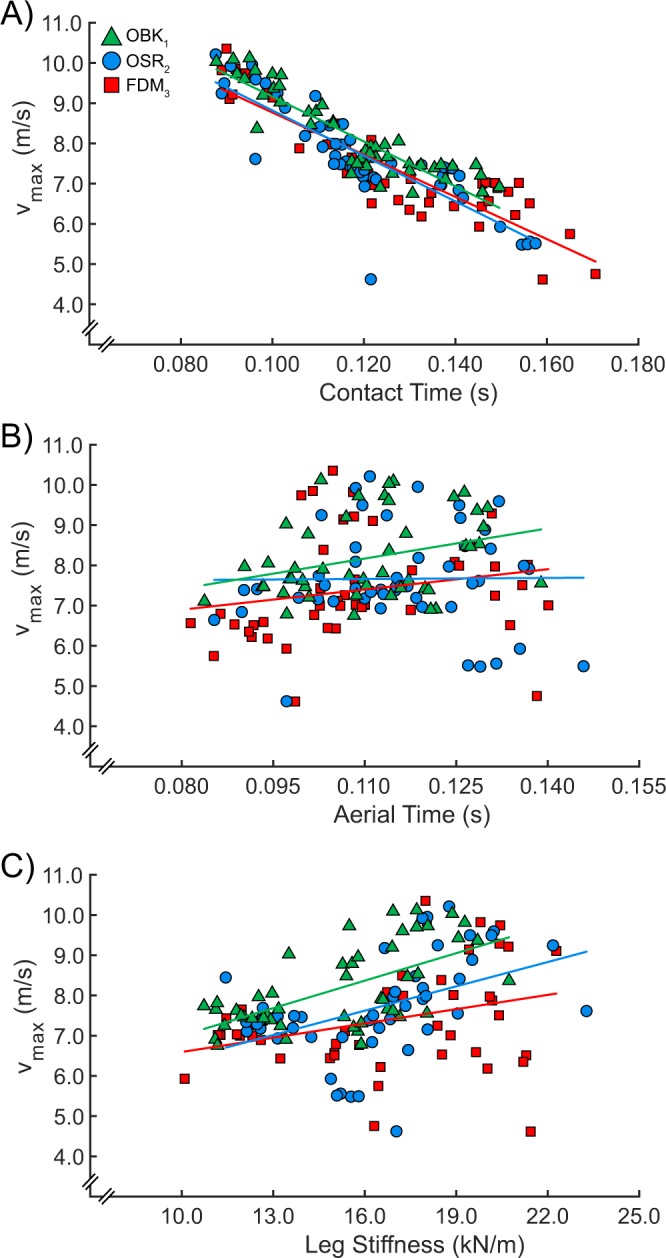
Maximum velocity (v_max_) as a function of (**A**) contact time, (**B**) aerial time, and (**C**) leg stiffness when using Ottobock 1E90 Sprinter (OBK_1_), Össur Cheetah Xtend (OSR_2_), and Freedom Innovations Catapult (FDM_3_), running-specific prosthetic models. Each individual data point represents the average value between the affected and unaffected legs for a single trial. Trend lines represent linear regressions for each prosthetic model. Coefficients of determination for linear regressions are shown in Table 2.

When accounting for differences in v_max_, we found that use of OSR_2_ resulted in 1.4% and 1.6% shorter ground contact times than use of OBK_1_ and FDM_3_ RSPs, respectively (β_1_ = 0.0027 and β_3_ = 0.0031, both p < 0.05; Fig. 4A, Table 2). However, there were no significant differences in contact times when athletes used OBK_1_ compared to FDM_3_ RSPs (p = 0.73). Use of OBK_1_ and OSR_2_ resulted in 3.7% and 5.7% longer aerial times than use of FDM_3_ RSPs, respectively (β_1_ = 0.0054 and β_2_ = 0.0083, both p < 0.05) and there were no significant differences in aerial times when athletes used OBK_1_ compared to OSR_2_ RSPs (p = 0.057; Fig. 4B, Table 2). Use of OBK_1_ resulted in 6.4% lower leg stiffness than use of OSR_2_ and FDM_3_ RSPs (β_2_ = 1.109 and β_3_ = 1.098, both p < 0.05) and there was no significant difference in leg stiffness between use of OSR_2_ and FDM_3_ RSPs (p = 0.97; Fig. 4C, Table 2).

**Figure 5:**
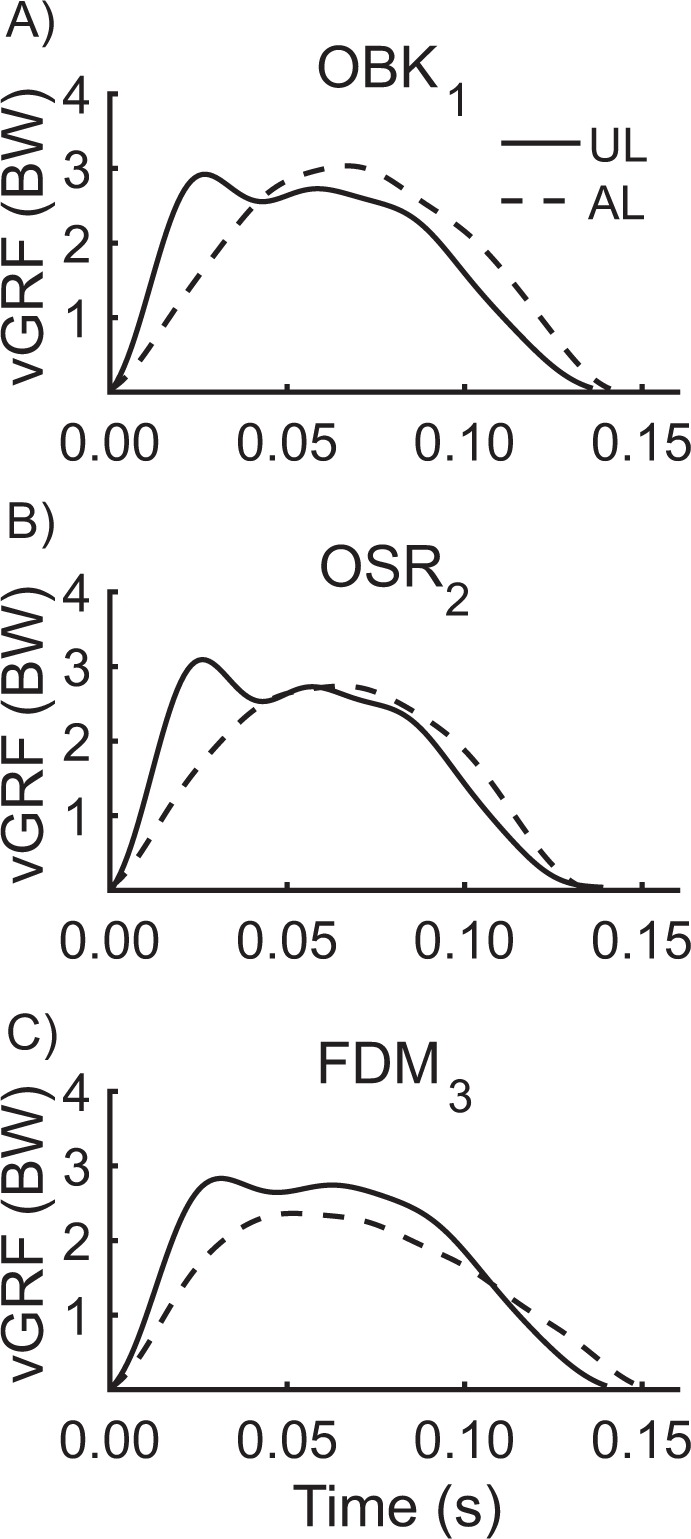
Representative vertical ground reaction force (vGRF) traces for the affected leg (AL) and unaffected leg (UL) when using (**A**) Ottobock 1E90 Sprinter (OBK_1_), (**B**) Össur Cheetah Xtend (OSR_2_), and (**C**) Freedom Innovations Catapult (FDM_3_) running-specific prosthetic models (recommended stiffness category and height) at maximal running velocity (OBK_1_ = 7.46 m/s, OSR_2_ = 7.43 m/s, FDM_3_ = 7.00 m/s).

We found that step frequency SI was less than zero when using all three RSPs (all p < 0.05; Table 3); on average, step frequency was 6.4% slower for the AL compared to UL. However, there were no differences in step frequency SI between RSP models (OBK_1_ vs. OSR_2_: p = 0.31; OBK_1_ vs. FDM_3_: p = 0.72; OSR_2_ vs. FDM_3_: p = 0.16). Stance average vertical GRF SI was less than zero across RSP conditions (all p < 0.05); on average stance average vertical GRFs were 6.2% lower for the AL compared to UL (Fig. 5, Table 3). However, use of OBK_1_ and OSR_2_ resulted in stance average vertical GRF SIs that were 0.078 and 0.105 more symmetric, respectively, compared to use of FDM_3_ RSPs (both p < 0.05). There was no significant difference in stance average vertical GRF SI between use of OBK_1_ and OSR_2_ RSPs (p = 0.16). Contact length SI was greater than zero when using all three RSPs (all p < 0.05); on average, contact lengths were 7.2% longer for the AL compared to UL. However, use of OSR_2_ resulted in contact length SIs that were 0.045 and 0.022 more symmetric compared to use of OBK_1_ and FDM_3_ RSPs, respectively (both p < 0.05), and use of FDM_3_ resulted in contact length SIs that were 0.023 more symmetric compared to use of OBK_1_ (p < 0.05).

**Table 3:** Average symmetry indices (SI) between the affected leg (AL) and unaffected leg (UL) for biomechanical variables when using Ottobock 1E90 Sprinter (OBK_1_), Össur Cheetah Xtend (OSR_2_), and Freedom Innovations Catapult (FDM_3_) running-specific prosthetic models. A positive SI means that the value for the AL is greater than the value for the UL, a negative SI means that the value for the UL is greater than the value for the AL, and an SI of zero indicates perfect symmetry between AL and UL. All SIs are significantly different than zero (p < 0.05).

	Average SI		
	OBK_1_	OSR_2_	FDM_3_
SI step frequency	−0.070	−0.079	−0.055
SI stance average vertical ground reaction force	−0.038	−0.030	−0.137
SI contact length	0.078	0.055	0.079
SI contact time	0.081	0.034	0.050
SI aerial time	0.070	0.131	0.068
SI leg stiffness	−0.216	−0.165	−0.127

We found that contact time SI was greater than zero when using all three RSPs (all p < 0.05); on average, ground contact times were 5.3% longer for the AL compared to UL. Use of OBK_1_ resulted in contact time SIs that were 0.047 and 0.031 more asymmetric than use of OSR_2_ and FDM_3_ RSPs, respectively (both p < 0.05). Further, use of OSR_2_ resulted in contact time SIs that were 0.016 more symmetric than use of FDM_3_ RSPs (p < 0.05). Aerial time SI was greater than zero when using all three RSPs (all p < 0.05); on average aerial times were 9.4% longer for the AL compared to UL. Use of OSR_2_ resulted in aerial time SIs that were 0.061 and 0.062 more asymmetric than use of OBK_1_ and FDM_3_ RSPs, respectively (both p < 0.05). There was no significant difference in aerial time SI between use of OBK_1_ and FDM_3_ RSPs (p = 0.79). Finally, leg stiffness SI was less than zero when using all three RSPs (all p < 0.05); on average, leg stiffness was 15.7% lower for the AL compared to UL. Use of OBK_1_ resulted in leg stiffness SIs that were 0.052 and 0.090 more asymmetric than use of OSR_2_ and FDM_3_ RSPs, respectively (both p < 0.05). There was no difference in leg stiffness SI between use of OSR_2_ and FDM_3_ RSPs (p = 0.18).

## Discussion

We reject our initial hypothesis because RSP model, but not stiffness or height, affects maximum running velocity in athletes with unilateral TTAs. Therefore, we only considered RSP model as a factor in determining the optimal configuration. Specifically, use of OBK_1_ resulted in the fastest v_max_, followed by use of OSR_2,_ and FDM_3_ RSPs. These results are in agreement with a previous study of running at 2.5–3.0 m/s for athletes with unilateral TTAs, which found that RSP model, but not stiffness or height, affects metabolic cost^20^. Thus, in addition to optimizing distance running performance (i.e. metabolic cost), results of the present study suggest that use of a J-shaped RSP model could also improve sprinting performance (i.e. v_max_) for athletes with unilateral TTAs compared to use of a C-shaped RSP.

There were biomechanical similarities when athletes with a TTA used OBK_1_ and OSR_2_ that were not present when they used FDM_3_ RSPs that may explain the differences in v_max_ elicited by use of different RSP models. Due to the inherent relationships between velocity and the biomechanical variables we investigated, we controlled for differences in velocity and determined the biomechanics elicited by use of different RSP models. When controlling for v_max_, use of J-shaped RSPs (OBK_1_ and OSR_2_) resulted in greater stance average vertical GRF compared to the C-shaped RSP (FDM_3_). Additionally, compared to use of FDM_3_, use of OBK_1_ and OSR_2_ RSPs resulted in slower step frequencies due to longer aerial times together with shorter (OBK_1_) or similar (OSR_2_) ground contact times. These biomechanical findings may be attributable to RSP shape; OBK_1_ and OSR_2_ are J-shaped RSPs while FDM_3_ is C-shaped. Compared to C-shaped RSPs, J-shaped RSPs have about 1% lower hysteresis and are wider^4,20,21^. The lower hysteresis from J-shaped RSPs could result in greater vertical CoM velocity at the end of the stance phase, and a longer aerial time for a given contact time, which would decrease step frequency. The wider geometry of J-shaped RSPs could enhance stability during running. When balance is perturbed, individuals with TTAs increase step frequency and decrease step length during walking, presumably to minimize the risk of falling^25^. It is possible that use of the narrower C-shaped RSPs resulted in greater medio-lateral instability that required athletes to run with increased step frequency compared to use of wider J-shaped RSPs. It is also possible that the alignment of the J-shaped RSPs enables a more vertical leg position during the stance phase compared to the C-shaped RSP. This could allow the leg to be more closely aligned with the GRF vector, resulting in a better effective mechanical advantage^26^ and greater stance average vertical GRFs. Further, non-amputees achieve faster running speeds by producing greater stance average vertical GRF^9^. Our results show that the ability to generate greater stance average vertical GRFs may also be important for athletes with a TTA to achieve faster running speeds, because use of J-shaped RSPs resulted in faster v_max_ and higher stance average vertical GRFs compared to use of a C-shaped RSP.

**Figure 6:**
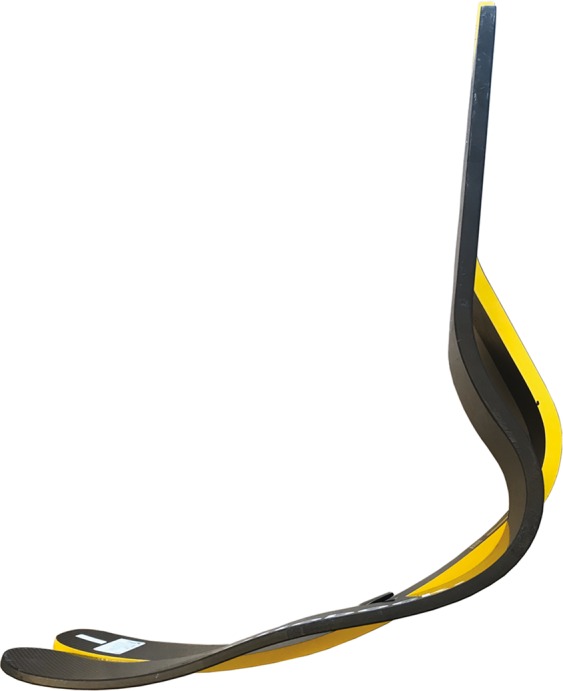
Lateral view of the J-shaped running specific prostheses (RSPs) showing differences in curvature. Ottobock 1E90 Sprinter (OBK_1_) is shown in front (black) and Össur Cheetah Xtend (OSR_2_) is shown behind (yellow).

We also found some biomechanical differences between use of the two different J-shaped RSPs (OBK_1_ and OSR_2_) that could explain differences in v_max_. Use of OBK_1_ resulted in lower stance average vertical GRFs and longer contact lengths than use of OSR_2_ RSPs when accounting for velocity. These differences were offset, and step lengths were similar when using OBK_1_ and OSR_2_ RSPs. Although step frequencies were similar when using OBK_1_ and OSR_2_ RSPs, contact times were longer (p < 0.05) and aerial times were numerically but not statistically shorter with use of OBK_1_ compared to OSR_2_ RSPs (p = 0.057). Additionally, use of OBK_1_ resulted in lower leg stiffness than use of OSR_2_ RSPs. These differences between RSP models could be due to variations in RSP geometry or alignment^4,20,21,27^ or differences in curvature between OBK_1_ and OSR_2_ RSPs (Fig. 6). Similar to alignment’s effect on stance average vertical GRF, perhaps the curvature of the OSR_2_ RSP enables a more vertical leg position during the stance phase compared to the OBK_1_ RSP. This could allow the leg to be better aligned with the GRF vector, resulting in greater stance average vertical GRF despite a shorter contact time. A more vertical leg position during initial ground contact would also lead to a smaller leg sweep angle, and thus shorter contact length and contact time. Additionally, a smaller leg sweep angle would decrease leg spring displacement during the stance phase (Eq. 4), which would increase leg stiffness, assuming peak vertical GRF is held constant. Therefore, differences in RSP geometry/curvature or alignment could explain why stance average vertical GRFs were lower, contact lengths and contact times were longer, and leg stiffness was lower with use of OBK_1_ compared to OSR_2_. Thus, future studies that determine the effects of RSP geometry/curvature and alignment are warranted to better understand the underlying biomechanical determinants of v_max_.

Our results partially support the hypothesis that the optimal RSP configuration would elicit more symmetric biomechanics. While considering differences in v_max_, RSP model did not affect step frequency symmetry, suggesting that step frequency symmetry may not be important for attaining faster v_max_. In contrast, RSP model did affect stance average vertical GRF symmetry. AL stance average vertical GRFs were, on average, 6.2% lower than UL stance average vertical GRFs with use of all three RSP models at v_max_. This finding is similar to a previous study that found AL stance average vertical GRFs were 7.7% lower than UL stance average vertical GRFs at v_max_^7^. In the present study, AL stance average vertical GRFs were 3.6%, 2.9%, and 12.2% lower than UL stance average vertical GRFs when using OBK_1_, OSR_2_, and FDM_3_ RSPs, respectively. Use of OBK_1_ and OSR_2_ elicited faster v_max_ than use of FDM_3_ RSPs and stance average vertical GRFs were more symmetric with use of OBK_1_ and OSR_2_ compared to FDM_3_ RSPs. This suggests that stance average vertical GRF symmetry may influence v_max_. RSP model also influenced contact length symmetry. AL contact lengths were, on average, 7.2% longer than UL contact lengths with use of all three RSP models at v_max_. This contrasts with a previous study that found no difference in contact length between the AL and UL when athletes with unilateral TTA ran at v_max_^7^. However, contact lengths were least symmetric with use of OBK_1_, suggesting that contact length symmetry may not influence v_max_. Use of the OBK_1_ RSP resulted in the most asymmetric and lowest average leg stiffness. Because use of the OBK_1_ RSP elicited the fastest v_max_, this suggests that lower average leg stiffness, but not leg stiffness symmetry, may influence v_max_ in athletes with unilateral TTAs. It is possible that lower leg stiffness results in a greater amount of time to generate force on the ground, which leads to faster v_max_. A previous study found that AL leg stiffness was 27% lower than UL leg stiffness at a maximum velocity of 9.5 m/s^6^. Similarly, we found that AL leg stiffness was 13–22% lower than UL leg stiffness at v_max_ across all configurations at an average v_max_ of 8.32 m/s. Overall, our results suggest that RSP configurations that result in more symmetric stance average vertical GRFs have the strongest influence on v_max_.

In the present study, we determined the effects of using fifteen different RSP configurations on v_max_ and the underlying biomechanics used to achieve these velocities. However, a potential limitation of this study is that some athletes used two different sockets to complete the protocol. Athletes used one socket for J-shaped RSPs (OBK_1_, OSR_2_) and a different socket for the C-shaped RSP (FDM_3_), which could have contributed to different amounts of residual limb movement within the socket, and potentially affected running biomechanics^21^. The mass of each RSP (OBK_1_: 675 g, OSR_2_: 750 g, FDM_3_: 510 g)^1–3^, in addition to the different sockets and attachments, may have affected the results. Specifically, different mass and therefore moment of inertia of the AL, could potentially affect leg swing time and step frequency. However, Grabowski *et al*.^7^ found no difference in v_max_ or in leg swing time of the AL when adding up to 300 g to the distal end of the RSP of athletes with unilateral TTAs compared to the same RSP with no added mass. We found that use of the FDM_3_ RSP resulted in 3.4% faster step frequencies than OBK_1_ and OSR_2_ RSPs yet use of the FDM_3_ RSP did not elicit the fastest v_max_.

Seven of the athletes involved this study typically use J-shaped RSPs for competition compared to three athletes who typically use C-shaped RSPs (Table 1). This familiarity could have affected our results. However, for nine athletes, irrespective of their usual RSP, J-shaped RSPs resulted in the fastest maximum velocity (Table 1). Only subject 2, who usually runs using a J-shaped RSP, had a faster v_max_ using the C-shaped FDM_3_. For all 3 of the subjects who usually run using a C-shaped RSP, use of the J-shaped RSPs resulted in a faster v_max_.

The spring-mass model has been used to calculate leg stiffness at running speeds ranging from 3.8 m/s^13^ to 8.7 m/s^28^ and up to 12.3 m/s^29^. Clark *et al*.^30^ found that the vertical GRF trace is not symmetric during ground contact for professional non-amputee sprinters, which suggests that the assumptions for calculating leg stiffness using the spring-mass model may be violated. We found that the vertical GRF trace for the UL is not symmetric during ground contact (Fig. 5), and thus the leg stiffness of the UL may not be accurately represented by the spring-mass model. These differences may limit our calculations of leg stiffness for the UL and the leg stiffness symmetry index comparing UL and AL leg stiffness. However, our findings are similar to those of McGowan *et al*.^5^, who calculated leg stiffness of the UL using the spring-mass model at speeds between 3.0 and 9.5 m/s.

Some athletes lowered themselves from the handrails onto the moving treadmill belt to initiate each trial while others stood on the treadmill and accelerated with the treadmill belt until it reached the desired velocity. Use of these different strategies is unlikely to influence v_max_ because the treadmill accelerates at 1.0 m/s^2^ and athletes used the same strategy for each RSP configuration. Additionally, the systematic trial order of progressively increasing velocity with each RSP configuration may have induced fatigue. To reduce any potential effects of fatigue, we allowed athletes rest *ad libitum*, and limited each day of testing to three sets of trials. The initial accommodation session was approximately four hours, but a longer accommodation period (multiple days/weeks) may have allowed athletes to reach a faster v_max_ for each RSP configuration. Future studies are needed to determine how athletes accommodate to RSPs, and how accommodation time relates to performance.

We found that RSP height did not significantly affect v_max_ (p = 0.11), but it is possible that changes of ±2 cm were not enough to significantly affect v_max_. However, ±2 cm changes in RSP height were noticeable by each athlete, and greater RSP height changes could have resulted in injury. The average maximum running velocity for all configurations in the present study (8.32 m/s) is similar to those reported previously for athletes with unilateral TTAs running on a treadmill (8.82 m/s and 8.75 m/s)^6,7^. Future studies are needed to investigate the effects of using different RSP configurations on joint mechanics and muscle activation patterns. These parameters could help to further explain differences in v_max_ among RSP configurations by revealing changes in effective mechanical advantage or ability to produce muscle force during the stance phase. Future studies should also investigate the effects of different RSP configurations on the start and acceleration phase of sprint races, to determine if the RSP configuration that allows for the fastest v_max_ is also the configuration that allows for a better start and acceleration, i.e. for a better racing performance.

## Conclusions

Athletes with unilateral TTAs reach different maximum running velocities when using different RSP models. Specifically, use of the J-shaped OBK_1_ resulted in the fastest v_max_, followed by use of the J-shaped OSR_2_, and C-shaped FDM_3_ RSPs. RSP stiffness and height do not have a significant effect on v_max_ in athletes with unilateral TTAs. While controlling for velocity, use of J-shaped versus C-shaped RSPs resulted in greater stance average vertical ground reaction forces, slower step frequencies, and longer step lengths. Stance average vertical ground reaction forces were less asymmetric, while contact time and leg stiffness were more asymmetric when using J-shaped versus C-shaped RSPs.

## Data Availability

All data generated and analyzed during the current study are presented in the main text, figures, and tables. An alternative data format is available from the corresponding author on reasonable request.
